# Supplementary material for: MaizeField3D: A curated 3D point cloud and procedural model dataset of field-grown maize from a diversity panel
Source: Plant Phenomics. 2025 Dec 15;8(1):100108. doi: 10.1016/j.plaphe.2025.100108 (PMC13109309; doi:10.1016/j.plaphe.2025.100108)
Supplement: Multimedia component 1 [file mmc1.pdf]

Supplementary Materials for: MaizeField3D: A Curated 3D Point Cloud and Procedural Model Dataset of Field-Grown Maize from a Diversity Panel

Elvis Kimara[1†], Mozhgan Hadadi[3†], Jackson Godbersen[3], Aditya Balu[2], Talukder Z. Jubery[2], Yawei Li[4,5,6], Adarsh Krishnamurthy[2,3,4], Patrick S. Schnable[4,5,6], and Baskar Ganapathysubramanian[2,3,4,*]

[1]Department of Computer Science, Iowa State University, Ames, USA
[2]Translational AI Research Center, Iowa State University, Ames, USA
[3]Department of Mechanical Engineering, Iowa State University, Ames, USA
[4]Plant Science Institute, Iowa State University, Ames, USA
[5]Interdepartmental Genetics and Genomics Graduate Program, Iowa State University, Ames, USA
[6]Department of Agronomy, Iowa State University, Ames, USA
[†]Equal Contribution
[*]Corresponding Author

Supplementary Materials

The Supplementary Materials provide visual examples and implementation details that complement the main text. Figure S.1 presents additional raw maize plant point clouds to further illustrate morphological diversity across accessions, and Figure S.2 shows additional segmented point clouds. Table S.1 lists the fixed color map used for organ labels (leaf indices 1–16 and stalk), with RGB and hex values. Table S.2 reports a runtime comparison of four downsampling strategies (Random, Voxel, FPS, and Poisson) at target sizes of 100k, 50k, and 10k points.

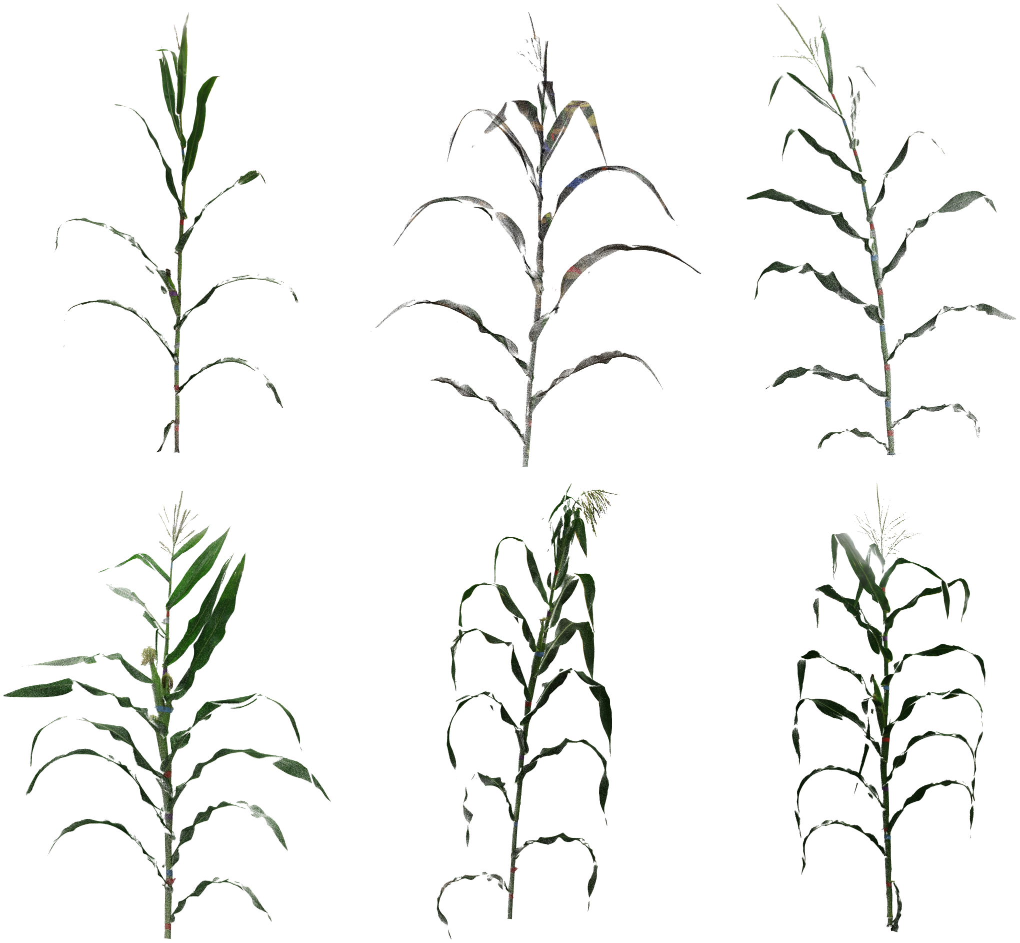

Figure S.1: *Additional maize plant point clouds from the MaizeField3D dataset, showcasing the original data. These examples further illustrate the morphological diversity observed across maize plant varieties.*

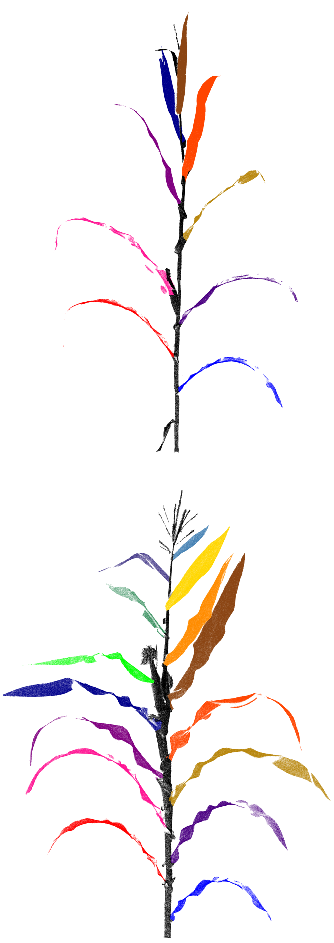

Figure S.2: *Additional examples of segmented maize plant point clouds from the dataset.*

Table S.1: *Color Coding for Plant Components*

Component	Color Name	RGB Values	Hex Code
Leaf 1	Bright Blue	[0, 0, 255]	#0000FF
Leaf 2	Bright Red	[255, 0, 0]	#FF0000
Leaf 3	Indigo	[75, 0, 130]	#4B0082
Leaf 4	Deep Pink	[255, 20, 147]	#FF1493
Leaf 5	Dark Goldenrod	[184, 134, 11]	#B8860B
Leaf 6	Purple	[128, 0, 128]	#800080
Leaf 7	Orange Red	[255, 69, 0]	#FF4500
Leaf 8	Dark Blue	[0, 0, 139]	#00008B
Leaf 9	Saddle Brown	[139, 69, 19]	#8B4513
Leaf 10	Bright Green	[0, 255, 0]	#00FF00
Leaf 11	Dark Orange	[255, 140, 0]	#FF8C00
Leaf 12	Sea Green	[46, 139, 87]	#2E8B57
Leaf 13	Gold	[255, 215, 0]	#FFD700
Leaf 14	Dark Slate Blue	[72, 61, 139]	#483D8B
Leaf 15	Steel Blue	[70, 130, 180]	#4682B4
Leaf 16	Brown	[165, 42, 42]	#A52A2A
Stalk	Black	[0, 0, 0]	#000000

Table S.2: *Runtime comparison (in seconds) of different downsampling strategies at three target sizes.*

Method	100k	50k	10k
Random	0.03 ± 0.01	0.03 ± 0.01	0.02 ± 0.01
Voxel	0.95 ± 0.26	1.01 ± 0.32	1.11 ± 0.34
FPS	107.57 ± 38.24	55.99 ± 19.59	11.26 ± 3.93
Poisson	108.30 ± 37.22	57.01 ± 19.95	11.58 ± 4.09
